# Insurance-related differences in high-intensity care utilization among older patients with cancer

**DOI:** 10.3389/fpubh.2026.1881472

**Published:** 2026-07-03

**Authors:** Rui Xu, Ru Meng, Zhenming Tang, Yuxin Qian, Fangli Su, Xinru Huang

**Affiliations:** 1 School of Management, Xuzhou Medical University, Xuzhou, China; 2 Medical Insurance Department, Xuzhou Cancer Hospital, Xuzhou, Jiangsu, China; 3 Institution of Chinese Health Modernization, Xuzhou Medical University, Xuzhou, Jiangsu, China

**Keywords:** clinical complexity, health equity, health insurance, healthcare utilization, multimorbidity, older adults

## Abstract

**Background:**

Health insurance is a key determinant of healthcare utilization, yet its influence on the allocation of high-intensity medical resources under varying levels of clinical need remains poorly understood.

**Methods:**

We conducted a retrospective observational study using administrative data from a tertiary hospital in China. The study included 12,916 hospitalized patients aged 60 years or older with a primary diagnosis of cancer in 2024. High-intensity care departments were classified using a multi-dimensional department-level proxy approach, in which quantitative indicators served as initial screening references, final classification was determined by senior clinician adjudication, and the resulting classification was further validated using available hospitalization-level resource-use indicators. Multivariable logistic regression and modified Poisson regression were used to estimate the association between insurance type and admission to high-intensity care departments. Interaction tests and subgroup analyses explored potential heterogeneity by age and multimorbidity.

**Results:**

Overall, 83.0% of patients were admitted to high-intensity care departments. Employee-insured patients had lower odds of admission than resident-insured patients (OR = 0.84, 95% CI: 0.74–0.94; RR = 0.98, 95% CI: 0.96–0.99). Multimorbidity was associated with higher odds of admission (OR = 1.32, 95% CI: 1.06–1.64). The formal interaction between insurance type and age was not significant (*p* = 0.096). Exploratory subgroup analyses suggested a potentially weaker association among patients with greater clinical complexity.

**Conclusion:**

Among older hospitalized patients with cancer, insurance type was associated with admission to high-intensity care departments. This association appeared to be weaker among patients with greater clinical complexity, suggesting that the influence of insurance-related factors may be attenuated when medical need becomes more pronounced. Exploratory analyses suggested a potentially weaker association among patients with greater clinical complexity; however, formal interaction tests did not provide statistically significant evidence of effect modification. These findings have implications for understanding equity in inpatient resource allocation within the context of ongoing health insurance payment reform and public hospital management in China.

## 1 Introduction

Healthcare utilization is shaped by the interplay between clinical need and institutional arrangements (1). Among these institutional factors, health insurance plays a central role in structuring access to care, influencing patterns of service use, and affecting the allocation of medical resources. In China, the coexistence of multiple insurance schemes, particularly employee insurance and resident insurance, has introduced substantial variation in benefit design, reimbursement levels, and financial protection, thereby contributing to disparities in healthcare utilization (2). Empirical evidence indicates that patients covered by more comprehensive insurance plans or with stronger financial protection tend to have higher utilization of inpatient and high-intensity care services (3).

Previous studies have extensively examined the relationship between insurance coverage and overall healthcare use, expenditure, and financial burden. Previous research has shown that admission to high-intensity care is influenced not only by patient severity but also by institutional practices, bed availability, and hospital policy (4). For example, capacity strain in ICUs can result in shorter but more resource intensive stays, and substantial between hospital variation persists even after adjusting for patient severity, highlighting the role of hospital level determinants (5). However, much less attention has been paid to whether insurance status is associated with access to high-intensity medical resources, such as admission to resource-intensive hospital departments. This issue is especially relevant in the context of ongoing health system reform in China, where improving efficiency and optimizing resource allocation remain important policy goals (6).

Importantly, the association between insurance and healthcare utilization may not be constant across patient populations. According to the Andersen behavioral model, healthcare use is influenced by predisposing characteristics, enabling resources, and need factors, with clinical need expected to become increasingly important as health conditions worsen (7). Among patients of advanced age or those with multimorbidity, healthcare utilization may therefore be driven more strongly by medical necessity than by institutional or socioeconomic factors (8).

Despite this theoretical expectation, empirical evidence remains limited regarding whether insurance-related differences persist under conditions of greater clinical complexity (9). In particular, it remains unclear whether differences in insurance coverage continue to be associated with admission to high-intensity care among older adults with cancer and multimorbidity (10).

To address this gap, the present study examined the association between insurance type and admission to high-intensity care departments among older hospitalized patients with cancer in China. We also explored whether this association varied by age and multimorbidity, thereby exploring whether insurance-related differences in utilization may vary as clinical need becomes more pronounced (11). Because direct patient-level measures of treatment intensity were unavailable, high-intensity care departments were defined using a multidimensional department-level proxy and validated with available hospitalization-level resource-use indicators (12). We further explored whether this association varied by age and multimorbidity, treating these analyses as exploratory assessments. By focusing on department-level resource-intensive inpatient care, this study provides preliminary evidence on insurance-related differences in inpatient care patterns and offers insights relevant to health equity and hospital resource allocation, particularly in the context of current health insurance and payment reforms.

## 2 Materials and methods

### 2.1 Data source and study population

This retrospective observational study used administrative data extracted from the Hospital Information System of a tertiary general hospital in eastern China. The dataset included hospitalized patients aged 60 years or older with a primary diagnosis of cancer in 2024. Data screening was conducted according to predefined eligibility criteria, including verification of key analysis variables and identification of repeated admissions. A total of 12,916 unique patients were included in the final analysis. No statistical imputation was performed.

The study was approved by the Ethics Committee of Xuzhou Medical University (No. XMU. MS. REC.2026.0612). Because this was a retrospective study using anonymized administrative data, the requirement for individual informed consent was waived by the ethics committee.

### 2.2 Variables

#### 2.2.1 Outcome variable

The outcome variable was admission to a high-intensity care department (yes/no). Because direct patient-level measures of treatment intensity, such as ICU admission, cancer stage, detailed treatment modality, functional status, and procedure-specific resource consumption, were not available in the administrative dataset, we used a department-level resource-intensity proxy. Therefore, this outcome should be interpreted as admission to a resource-intensive department rather than direct patient-level receipt of intensive treatment.

Departments were classified through a multi-dimensional department-level proxy approach. Quantitative indicators, including mean hospitalization cost, department-level case mix index (CMI), surgery/procedure rate, and mean length of stay, were used as initial screening and classification references. The 75th percentile of mean hospitalization cost was used as a descriptive screening reference rather than a strict deterministic cutoff. Final classification was determined by senior clinician adjudication, considering quantitative indicators, department function, typical oncology care pathways, procedural intensity, and clinical plausibility. Thus, departments were not required to exceed a single threshold on every indicator to be classified as high-intensity. This method aligns with prior research that uses composite measures constructed from multiple quantitative indicators with expert panel review to ensure validity (13) and feasibility in health services measurement (14).

The final classification identified 16 high-intensity departments and 12 non-high-intensity departments. To evaluate the construct validity of this proxy, we compared available hospitalization-level resource-use indicators between high-intensity and non-high-intensity departments, including total hospitalization cost, reimbursement amount, CMI, LOS, and surgery/procedure rate. The complete department list and validation indicators are provided in Table 1. The validity of the final department classification was subsequently assessed using hospitalization-level resource-use indicators across all included patients, with the results presented in Table 2.

**Table 1:** Department classification by mean hospitalization cost, CMI, and resource indicators.

Department	*N*	Mean cost (CNY)	CMI	Mean LOS (days)	Surgery rate (%)	Classification
ICU	29	61,265	4.99	9.83	100.0	High-intensity
Neurosurgery	6	52,224	2.48	19.17	66.7	High-intensity
Orthopedics	18	27,561	1.65	13.56	94.4	High-intensity
Emergency	1	27,457	1.32	13.00	100.0	High-intensity
Interventional radiology I	48	21,779	2.96	11.19	79.2	High-intensity
Hepatobiliary surgery	382	20,206	1.80	9.99	75.4	High-intensity
Head and neck surgery	142	17,813	1.26	10.07	92.3	High-intensity
Radiation oncology	2,706	17,600	1.44	14.69	67.4	High-intensity
Thoracic surgery I	775	16,861	1.30	7.43	76.0	High-intensity
Gastrointestinal surgery	648	16,338	1.44	13.23	87.2	High-intensity
Nuclear medicine	23	15,365	2.55	3.96	95.7	High-intensity
Thoracic surgery II	1,003	14,648	1.10	8.82	61.1	High-intensity
Cardiology	24	13,132	1.15	7.54	54.2	High-intensity
Minimally invasive surgery	30	13,052	1.18	5.77	96.7	High-intensity
Gynecologic oncology	206	12,938	1.31	7.11	84.0	High-intensity
Medical oncology	4,678	10,405	1.06	6.69	62.0	High-intensity
Interventional radiology II	66	13,433	2.70	7.33	43.9	Non-high-intensity
Oral surgery	11	13,397	1.08	15.18	72.7	Non-high-intensity
Urology	577	13,267	1.10	8.01	79.0	Non-high-intensity
Integrative medicine	470	11,223	1.14	8.43	68.5	Non-high-intensity
Respiratory medicine	146	11,178	1.16	10.58	59.6	Non-high-intensity
Interventional ultrasound	5	10,268	1.26	4.20	60.0	Non-high-intensity
Nephrology	5	10,075	0.86	13.00	20.0	Non-high-intensity
Gastroenterology	67	9,528	0.76	7.93	85.1	Non-high-intensity
Geriatrics	6	9,184	0.92	8.83	16.7	Non-high-intensity
Neurology	6	8,982	0.82	9.33	0.0	Non-high-intensity
Breast surgery	821	8,495	0.94	5.31	95.9	Non-high-intensity
Traditional Chinese medicine	17	6,493	0.67	8.24	0.0	Non-high-intensity

**Table 2:** Validation of department classification by patient-level and department-level indicators.

Indicator	High-intensity (*n* = 10,719)	Non-high-intensity (*n* = 2,197)	*p-*value	Ratio
Mean total cost (CNY)	14,206	10,710	<0.001	1.33
Median total cost (CNY)	9,193	7,453	<0.001	—
CMI	1.33	0.98	<0.001	—
Mean reimbursement (CNY)	8,772	6,785	<0.001	1.29
Mean LOS (days)	9.57	7.29	<0.001	1.31
Median LOS (days)	7 (4–11)	5 (3–9)	<0.001	—

#### 2.2.2 Key independent variable

The primary independent variable was insurance type, categorized as employee insurance or resident insurance.

#### 2.2.3 Covariates

**Figure 1:**
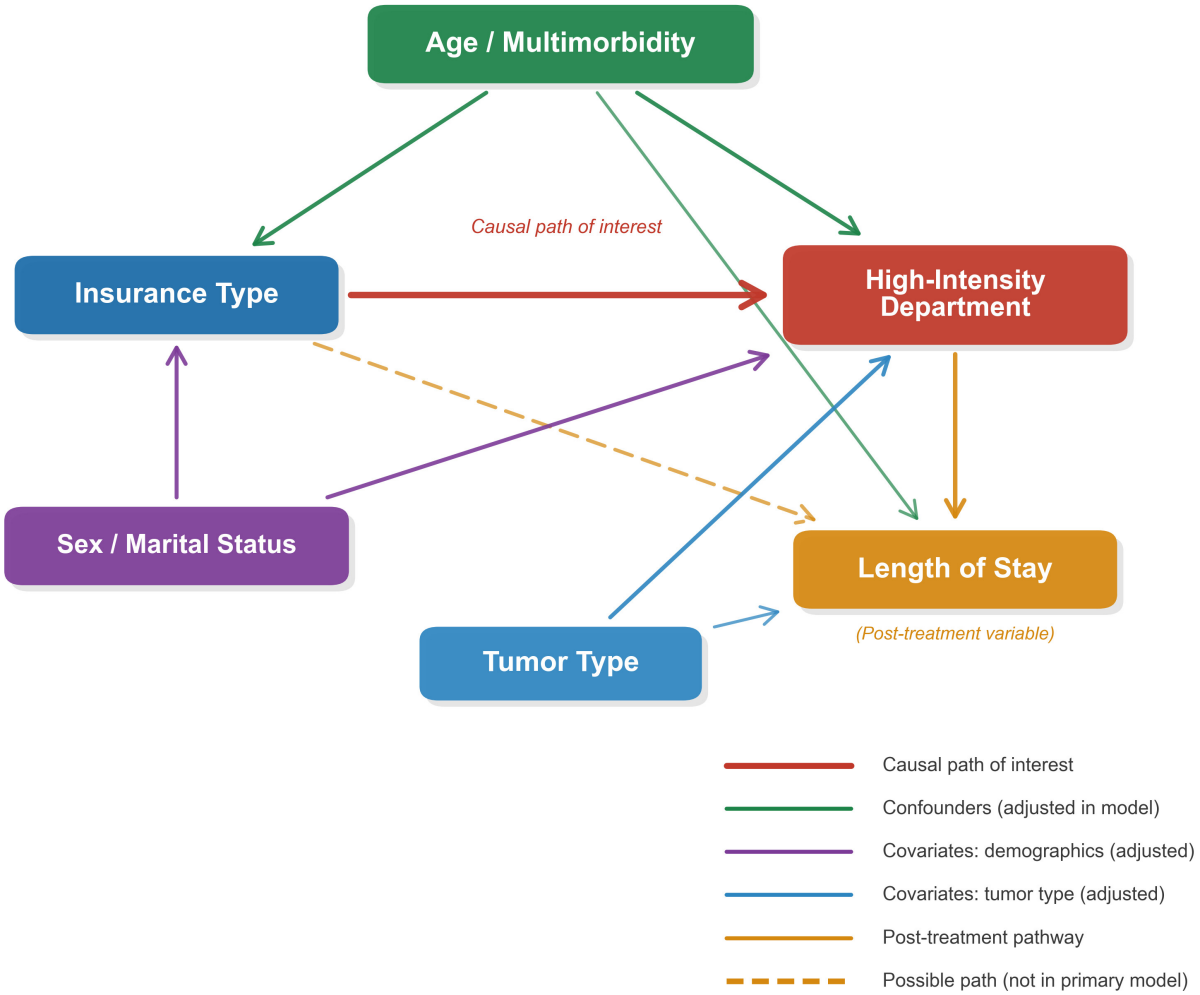
DAG of the hypothesized causal framework.

Covariates were selected based on prior literature and a directed acyclic graph (DAG) framework (Figure 1). The DAG identified the following variables as confounders requiring adjustment: age group (60–69, 70–79, and 80 years or older), sex (male/female), marital status (married, unmarried/divorced/widowed, other), multimorbidity status, and tumor type.

Multimorbidity was assessed using the Charlson Comorbidity Index (CCI), calculated from ICD-10 diagnostic codes recorded during the hospitalization. Cancer-related components were excluded from the CCI calculation to avoid overestimation of comorbidity burden in this cancer-specific population. Patients with a modified CCI score of 2 or higher were classified as having multimorbidity.

Tumor types were categorized according to 3-character ICD-10 codes. The 10 most frequent tumor categories were retained as individual categories, and the remainder were grouped as “Other.” The reference category was C34 (lung cancer), the most frequent tumor type in this population.

### 2.3 Statistical analysis

Baseline characteristics were summarized using frequencies and percentages for categorical variables and medians with interquartile ranges (IQR) for continuous variables. Differences between groups were assessed using chi-square tests for categorical variables and Wilcoxon rank-sum tests for continuous variables. Multivariable logistic regression was used to estimate the association between insurance type and admission to high-intensity care departments. The model adjusted for age group, sex, marital status, multimorbidity, and tumor type. Adjusted odds ratios (ORs) with 95% confidence intervals (CIs) were reported. Robust (Huber-White) standard errors were used to account for potential heteroscedasticity. Because the outcome was defined by department classification, department-level clustering was not incorporated into the primary regression models. However, department-specific practice variation remains a potential source of residual correlation.

The interaction between insurance type and age group was assessed using a likelihood ratio test comparing nested logistic regression models (with and without the interaction term). Subgroup analyses were conducted separately by age group (60–69, 70–79, ≥80) and multimorbidity status (CCI < 2 vs. CCI ≥ 2). Average marginal effects (AMEs) were estimated to quantify age-stratified differences in predicted probabilities of high-intensity admission associated with insurance type. These analyses are explicitly exploratory; the study was not powered for formal interaction testing.

Two pre-specified sensitivity analyses were conducted: (1) re-estimation of the logistic regression model with log-transformed LOS included as an additional covariate; (2) comparison of effect estimates between the logistic and modified Poisson models to assess the impact of the high-prevalence outcome on effect magnitude.

Multicollinearity was assessed using variance inflation factors (VIF) values below 5 were considered acceptable. Model discrimination was evaluated using the area under the receiver operating characteristic curve (AUC). Model discrimination was evaluated using AUC. Because the objective was estimation of adjusted associations rather than outcome prediction, model discrimination was considered a supplementary diagnostic measure. The estimand of interest is the adjusted association between insurance type and the probability of admission to a high-intensity care department, conditional on measured covariates. This is an associational estimand: we estimate whether insurance type is a statistical predictor of department allocation after accounting for observed confounders, without claiming that insurance type causally determines allocation.

All analyses were conducted using Python 3.11 with statsmodels (version 0.14) for regression modeling. Statistical significance was defined as *p* < 0.05 (two-tailed).

## 3 Results

### 3.1 Baseline characteristics

Baseline characteristics differed by admission status, particularly in insurance type, age group, sex, and marital status.

A total of 12,916 patients were included in the analysis, of whom 10,719 (83.0%) were admitted to high-intensity care departments. Table 3 summarizes the baseline characteristics of the study population according to admission status.

**Table 3:** Baseline characteristics of the study population by admission to high-intensity care departments.

Variable	Non-high-intensity (*n* = 2,197)	High-intensity (*n* = 10,719)	*p*-value
Insurance type			<0.001
Resident insurance (ref.)	1,168 (53.16%)	6,818 (63.61%)	
Employee insurance	1,029 (46.84%)	3,901 (36.39%)	
Age group			<0.001
60–69 (ref.)	1,175 (53.48%)	5,332 (49.74%)	
70–79	779 (35.46%)	4,392 (40.97%)	
≥80	243 (11.06%)	995 (9.28%)	
Multimorbidity (CCI)			<0.001
CCI < 2 (ref.)	2035 (92.63%)	9,557 (89.16%)	
CCI ≥ 2	162 (7.37%)	1,162 (10.84%)	
Sex			<0.001
Female (ref.)	1,221 (55.58%)	4,288 (40.00%)	
Male	976 (44.42%)	6,431 (60.00%)	
Marital status			<0.001
Married (ref.)	1,665 (75.79%)	8,665 (80.84%)	
Unmarried/divorced/widowed	19 (0.86%)	42 (0.39%)	
Other	513 (23.35%)	2012 (18.77%)	
Length of stay (days)	5 (3–9)	7 (4–11)	<0.001

Values are *n* (%) for categorical variables and median (IQR) for continuous variables. *p*-values from chi-square test (categorical) or Wilcoxon rank-sum test (continuous). Reference groups labeled with (ref.).

Significant differences were observed between the two groups with respect to insurance type, age group, sex, and marital status (all *p* < 0.05). Patients admitted to high-intensity care departments were more likely to be covered by resident insurance than those not admitted to such departments (63.61% vs. 53.16%, *p* < 0.001). The proportion of patients with multimorbidity (CCI ≥ 2) was higher among those admitted to high-intensity departments (10.84% vs. 7.37%, *p* < 0.001). Median length of stay was longer among patients admitted to high-intensity care departments than among those admitted to non-high-intensity departments (7 (4–11) days vs. 5 (3–9) days, *p* < 0.001).

### 3.2 Multivariable regression analysis

**Figure 2:**
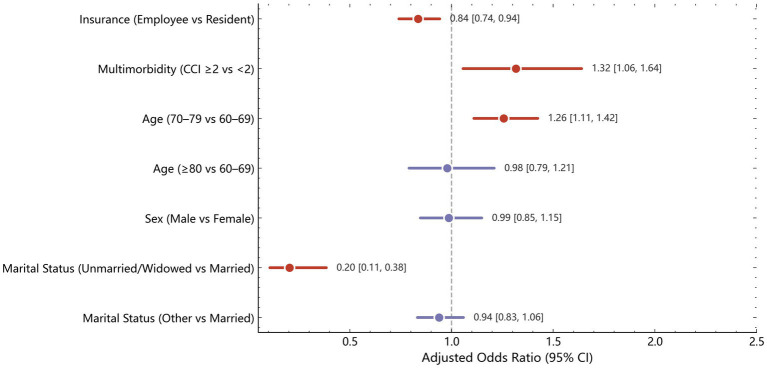
Adjusted odds ratios from the primary logistic model.

In multivariable logistic regression analyses (Figure 2; Table 4), insurance type was significantly associated with admission to high-intensity care departments. After adjustment for covariates (excluding LOS), patients covered by employee insurance had a lower likelihood of admission than those covered by resident insurance (OR = 0.84, 95% CI: 0.74–0.94, *p* = 0.003). On the risk-ratio scale, the corresponding risk ratio was 0.98 (95% CI: 0.96–0.99, *p* = 0.003), indicating that employee-insured patients had approximately 2% lower probability of admission to high-intensity departments. The difference between the OR (16% lower odds) and RR (2% lower risk) illustrates the well-known non-collapsibility of odds ratios when the outcome is common.

**Table 4:** Multivariable logistic regression analysis of factors associated with high-intensity care departments utilization.

Variable	OR (95% CI)	*P* value	RR (95% CI)	*P* value
Insurance type (employee vs. resident, ref.)	0.84 (0.74–0.94)	0.003	0.98 (0.96–0.99)	0.003
Multimorbidity (CCI ≥ vs. < 2, ref.)	1.32 (1.06–1.64)	0.014	1.03 (1.01–1.05)	0.009
Age 70–79 vs. 60–69 (ref.)	1.26 (1.11–1.42)	<0.001	1.03 (1.01–1.04)	<0.001
Age ≥80 vs. 60–69 (ref.)	0.98 (0.79–1.21)	0.842	1.00 (0.97–1.02)	0.820
Sex (male vs. female, ref.)	0.99 (0.85–1.15)	0.860	1.00 (0.99–1.01)	0.988
Unmarried/divorced/widowed vs. married (ref.)	0.20 (0.11–0.38)	<0.001	0.77 (0.65–0.91)	0.002
Other vs. married (ref.)	0.94 (0.83–1.06)	0.301	0.99 (0.98–1.01)	0.211

Primary model = logistic regression without LOS (*N* = 12,916; AUC = 0.815; Pseudo R-squared = 0.296; Mean VIF = 1.04). Modified Poisson model with robust standard errors. Both models adjusted for age, sex, marital status, multimorbidity, and tumor type. Reference groups indicated with (ref.). Tumor type coefficients omitted for brevity (reference = C34 lung cancer).

Multimorbidity was positively associated with admission (OR = 1.32, 95% CI: 1.06–1.64, *p* = 0.014; RR = 1.03, 95% CI: 1.01–1.05, *p* = 0.009). Age 70–79 was associated with higher odds compared with age 60–69 (OR = 1.26, 95% CI: 1.11–1.42, *p* < 0.001), whereas the association for age 80 or older was not significant (OR = 0.98, 95% CI: 0.79–1.21, *p* = 0.842). Sex was not significantly associated with admission (OR = 0.99, *p* = 0.860).

Multicollinearity diagnostics showed no evidence of substantial collinearity among the independent variables (mean VIF = 1.04, all individual VIF < 1.12). The model showed good discrimination (AUC = 0.815), although discrimination should not be interpreted as the primary criterion for evaluating an explanatory model (Figure 2).

Figure 2 displays the forest plot of adjusted odds ratios from the primary logistic regression model. Among the covariates shown, employee insurance was associated with lower odds of admission to high-intensity care departments compared with resident insurance. Multimorbidity and age 70–79 years were associated with higher odds of admission, whereas age ≥80 years and sex were not statistically significant. Patients who were unmarried, divorced, or widowed had substantially lower odds of admission compared with married patients.

### 3.3 Exploratory heterogeneity analysis

The formal interaction between insurance type and age group was assessed using a likelihood ratio test. The interaction term was not statistically significant (*p* = 0.096), indicating no strong evidence that the insurance-admission association differs across age groups at the conventional significance level. Despite the non-significant interaction, we present exploratory subgroup results to describe the data pattern while emphasizing that these do not constitute confirmatory evidence of heterogeneity.

**Figure 3:**
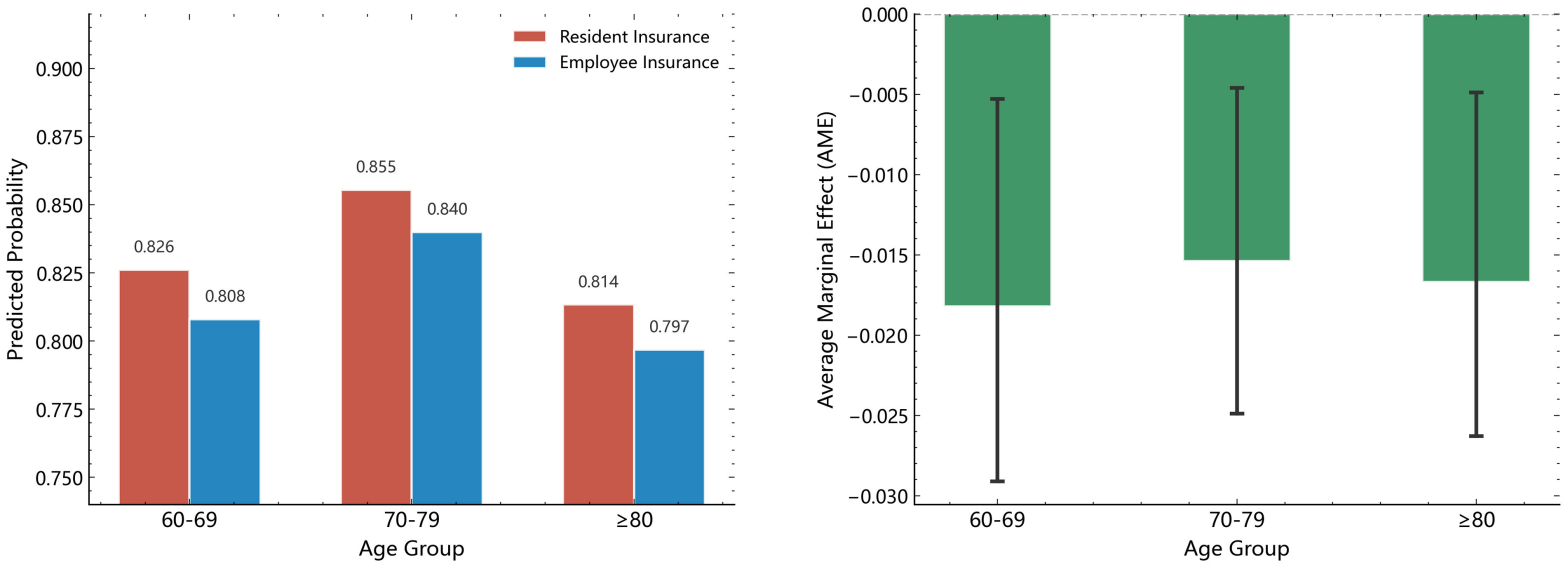
Predicted probabilities and average marginal effects.

Figure 3 displays predicted probabilities of admission by insurance type across age groups, along with average marginal effects (AME). Across all age groups, patients covered by resident insurance had slightly higher predicted probabilities of admission. The AME of employee insurance (relative to resident insurance) was −0.018 (95% CI: −0.029 to −0.005, *p* = 0.005) in the 60–69 group, −0.015 (95% CI: −0.025 to −0.005, *p* = 0.005) in the 70–79 group, and −0.017 (95% CI: −0.026 to −0.005, *p* = 0.004) in the 80 + group.

The relatively similar AME magnitudes across age groups (ranging from −0.015 to −0.018) suggest that the insurance-associated probability difference does not clearly diminish with age in these data, despite the non-significant interaction. The predicted probabilities show that admission rates are high across all strata (approximately 80–86%), with the insurance-related gap remaining modest throughout.

### 3.4 Subgroup analysis

**Figure 4:**
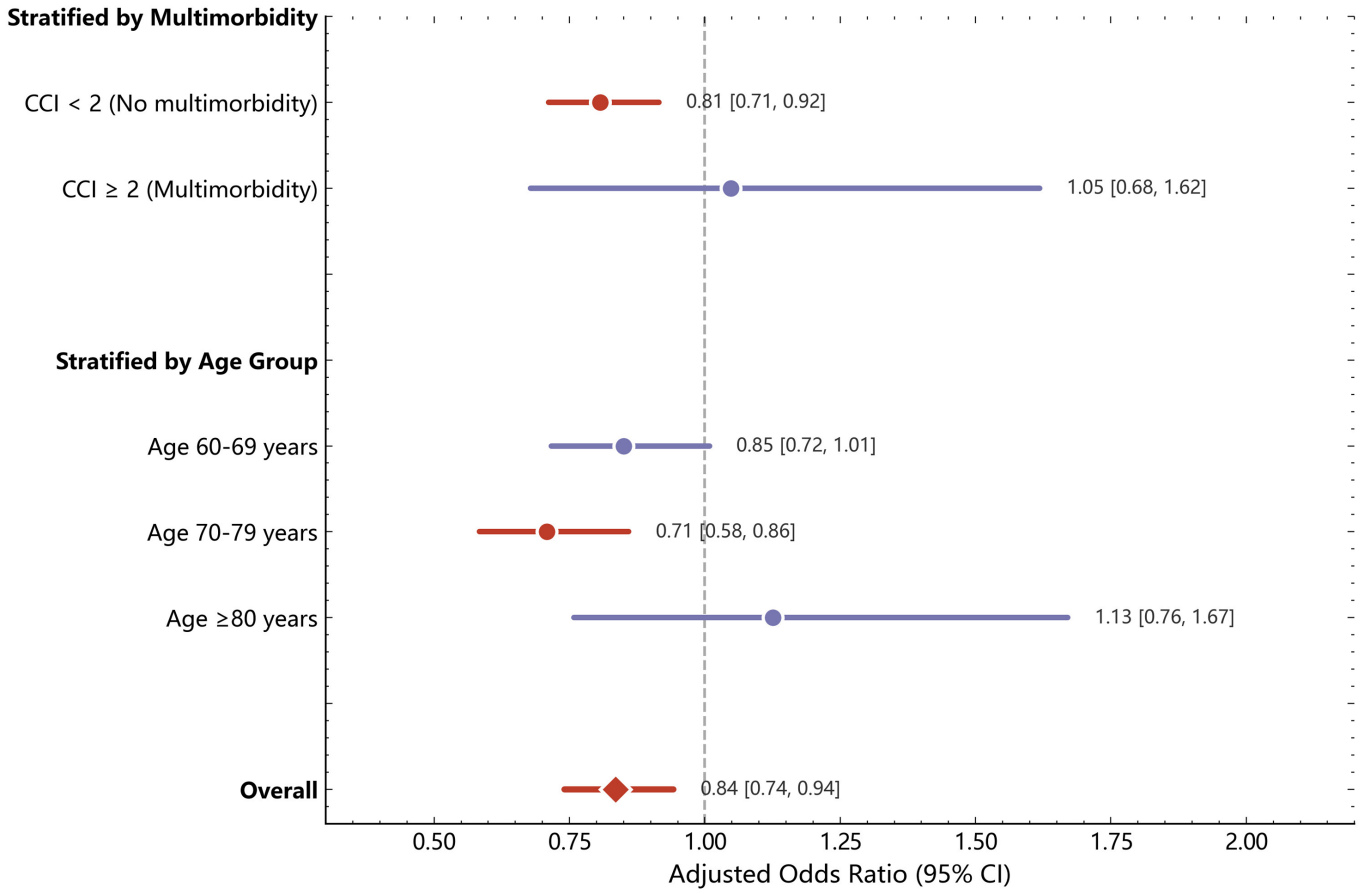
Stratified analysis by age and multimorbidity.

Subgroup analyses further explored the insurance-admission association across clinical strata (Figure 4). Among patients without multimorbidity (*n* = 11,592), employee insurance was associated with a significantly lower likelihood of admission (OR = 0.81, 95% CI: 0.71–0.92, *p* < 0.001). Among patients with multimorbidity (*n* = 1,324), no statistically significant association was observed (OR = 1.05, 95% CI: 0.68–1.62, *p* = 0.833).

Age-stratified analyses showed that the insurance association was strongest among patients aged 70–79 (OR = 0.71, 95% CI: 0.58–0.86, *p* < 0.001), borderline among those aged 60–69 (OR = 0.85, 95% CI: 0.72–1.01, *p* = 0.062), and non-significant among those aged 80 or older (OR = 1.13, 95% CI: 0.76–1.67, *p* = 0.556).

The formal interaction test between insurance type and age group was not statistically significant, indicating no confirmed effect modification. The observed pattern, with the largest association in the 70–79 age group rather than the youngest group, combined with wide confidence intervals in smaller subgroups, limits interpretability. Accordingly, these findings are presented as exploratory and hypothesis-generating observations, rather than as definitive evidence of heterogeneity across age strata.

### 3.5 Sensitivity and robustness analysis

To assess the robustness of the main findings, two additional analyses were performed.

First, the logistic regression model was re-estimated including log-transformed length of stay (LOS) as a covariate. The estimated effect of insurance type remained virtually unchanged (OR = 0.84, 95% CI: 0.74–0.94, *p* = 0.003), while LOS itself was positively associated with admission, though modest in magnitude (OR = 1.10, 95% CI: 1.01–1.18, *p* = 0.023).

**Figure 5:**
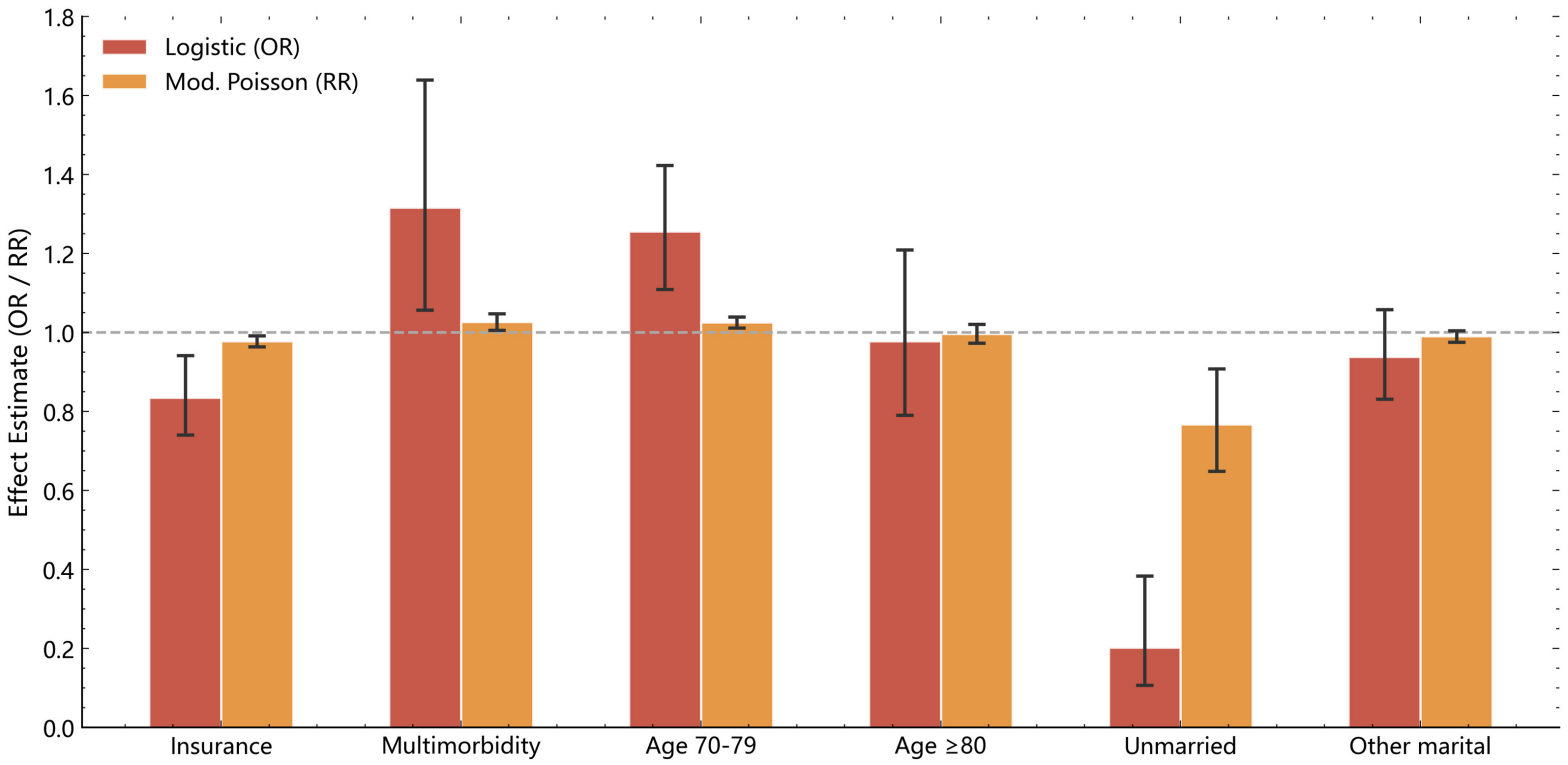
Effect estimates from logistic and modified Poisson model.

Second, the modified Poisson regression model produced effect estimates consistent in direction and significance. The corresponding risk ratio for insurance (RR = 0.98, *p* = 0.003) was smaller than the OR, reflecting the well-known overestimation of relative differences by ORs in the context of high-prevalence outcomes. Figure 5 presents a comparison of the effect estimates from both approaches.

Overall, the consistency of findings across models and specifications reinforces the robustness of the observed association between insurance type and admission to high-intensity care departments.

## 4 Discussion

This study examined insurance-related differences in admission to resource-intensive inpatient departments among older hospitalized patients with cancer at a single tertiary hospital in eastern China. After adjusting for demographic and clinical characteristics, employee-insured patients were less likely to be admitted to high-intensity care departments than resident-insured patients, with lower odds in the logistic regression model (OR = 0.84) and a modestly lower probability on the risk-ratio scale (RR = 0.98). Although statistically significant, the absolute difference was small, suggesting that the observed association represents a modest effect. The association remained consistent across alternative modeling approaches and sensitivity analyses, including the inclusion or exclusion of length of stay. Exploratory subgroup analyses suggested potential variation across clinical strata, particularly by multimorbidity status; however, the formal interaction test was not statistically significant (*p* = 0.096), and these findings should be interpreted as hypothesis-generating rather than confirmatory evidence of heterogeneity. Overall, these results provide preliminary evidence that insurance-related differences in admission to resource-intensive departments may persist even within a population characterized by high healthcare needs.

The observed association is broadly consistent with prior studies showing that insurance coverage influences healthcare utilization, inpatient service use, and medical expenditures. Previous studies in China have documented differences in healthcare utilization between and within insurance schemes (15), as well as associations between health insurance and outpatient or inpatient service use among middle-aged and older adults (16, 17). Similar associations between insurance type or insurance status and healthcare utilization have also been reported in other health systems and disease populations (18, 19). In China, disparities in healthcare utilization across insurance schemes have been documented even under the framework of universal health coverage. Evidence from resident insurance integration suggests that insurance scheme design may continue to affect health service equity (20). Studies on the redistributive effects of China’s urban–rural resident basic medical insurance and broader health insurance reforms further indicate that differences in financial protection remain relevant to equity in healthcare access and utilization (21, 22). Rural urban differences in healthcare use and expenditures among older adults also suggest that universal insurance coverage does not fully eliminate disparities in service utilization (23). Among cancer populations, socioeconomic and insurance-related differences have also been reported in inpatient treatment, healthcare use, and financial burden. A population-based study of lung cancer patients in China reported disparities in inpatient treatment and expenditures under tiered social health insurance (24). Cancer-related financial toxicity and financial burden have been identified as important challenges affecting patients with cancer in China (25, 26). In addition, older patients with cancer often require integrated care across the care continuum, highlighting the importance of examining resource-intensive inpatient care in this clinically vulnerable population (27). Our study extends this literature by focusing specifically on admission to resource-intensive inpatient departments among older hospitalized patients with cancer, highlighting patterns of insurance-related differences within a clinically vulnerable population.

Several plausible explanations may account for the observed association. Differences in benefit design, reimbursement policies, and financial protection across insurance schemes may shape outpatient management, healthcare-seeking behavior, treatment decisions, and the timing of hospital admission. Financial protection under multi-tiered health financing systems may influence healthcare utilization, particularly among vulnerable groups (28). In addition, healthcare-seeking behavior and service use may be shaped by the organization of the healthcare delivery system, including hierarchical medical care and access to appropriate services before hospitalization (29–31). In the context of cancer care, financial burden and insurance-related coverage may influence patients” care trajectories prior to hospitalization. For example, variations in outpatient access, continuity of care, early detection, and financial protection may affect the clinical condition at hospital presentation. However, information on cancer stage, disease severity, treatment intent, functional status, outpatient utilization, referral pathways, and patient preferences was unavailable, and these mechanisms could not be directly assessed; thus, they should be interpreted as plausible hypotheses rather than empirically demonstrated causal pathways.

Patient-level triage and department allocation also play an important role. Admission to a resource-intensive department is not determined solely by insurance type or patient-level medical need; it may also reflect tumor type, surgical candidacy, expected treatment pathway, bed availability, referral patterns, and department-specific practice norms. Utilization of intensive and high-acuity care has been shown to vary across hospitals and departments even after accounting for patient characteristics, suggesting that institutional and organizational factors contribute to allocation decisions (32, 33). More broadly, the equity and efficiency of healthcare resource allocation may differ across healthcare settings and regions, further supporting the need to interpret resource-intensive department admission as a system- and department-level allocation outcome (34). In this sense, the outcome captures a department-allocation process shaped by both patient characteristics and hospital practice patterns. Therefore, the observed insurance-related difference should be interpreted as an association with admission to resource-intensive departments rather than as direct evidence of differential treatment intensity at the individual patient level.

Exploratory subgroup analyses suggested that the insurance-admission association may vary by clinical complexity. Although formal interaction tests did not demonstrate statistically significant effect modification, the association appeared weaker among patients with multimorbidity (35, 36). This pattern is conceptually compatible with the Andersen behavioral model, which posits that healthcare utilization is influenced by predisposing, enabling, and need factors, with clinical need becoming more influential as patient complexity rises (37, 38). Confidence intervals were wide, the multimorbidity subgroup was smaller than the non-multimorbidity subgroup, and the study was not powered for formal heterogeneity testing; these findings should therefore be interpreted as hypothesis-generating rather than confirmatory evidence of effect modification.

The use of admission to high-intensity care departments as a proxy for treatment intensity requires careful interpretation. Direct patient-level measures such as ICU admission, cancer stage, detailed treatment modality, functional status, and procedure-specific resource use were unavailable. Instead, a multidimensional department-level classification combining quantitative indicators and senior clinician adjudication was used, providing construct validity at the department level. Nevertheless, this proxy reflects allocation to departments with higher average resource intensity, rather than direct patient-level treatment intensity. Some patients in high-intensity departments may have received lower-intensity care, and some patients in non-high-intensity departments may have required intensive management. Future studies should incorporate patient-level measures, including procedure-specific resource use, cancer stage, treatment modality, and surgical candidacy, to better distinguish department-level allocation from patient-level treatment intensity.

Several limitations should be considered when interpreting these findings. First, important clinical variables, including surgical candidacy, disease severity, and functional status, were unavailable, leaving the possibility of residual confounding. Second, this study was conducted in a single tertiary hospital, which may limit the generalizability of the findings to other healthcare settings. In addition, patients were clustered within hospital departments, and admission decisions may partly reflect department-specific practice patterns, organizational norms, or resource availability. Because the analysis did not explicitly account for department-level clustering, residual within-department correlation may have led to modest underestimation of standard errors. Future studies with larger numbers of departments and multicenter data could apply multilevel modeling approaches to better distinguish patient-level and department-level influences on resource allocation. Finally, the observational design precludes causal inference, and the observed associations should not be interpreted as evidence that insurance type directly determines admission to high-intensity care departments (39). Therefore, findings should be interpreted as differences in admission to resource-intensive departments rather than direct differences in patient-level receipt of intensive treatment.

Despite these limitations, this study contributes to the literature by examining insurance-related differences in the utilization of resource-intensive inpatient services among older hospitalized patients with cancer. By focusing on high-intensity care departments rather than overall healthcare use, the study provides additional evidence regarding healthcare equity and hospital resource allocation (40, 41). These findings highlight the importance of examining equity in resource allocation across insurance groups and suggest that future reforms should consider not only overall healthcare utilization but also the distribution of resource-intensive services among clinically vulnerable populations (42, 43). Further efforts to improve equity may require attention to the entire continuum of care, including outpatient management, early intervention, and access to healthcare services before hospitalization.

## 5 Conclusion

Insurance type was associated with admission to high-intensity care departments among older hospitalized patients with cancer. Patients covered by employee insurance were less likely to be admitted to high-intensity care departments than those covered by resident insurance after adjustment for measured covariates. Exploratory analyses suggested a potentially weaker association among patients with greater clinical complexity; however, formal interaction tests did not provide statistically significant evidence of effect modification. Given the observational design and the use of a department-level proxy for treatment intensity, these findings should be interpreted as evidence of association rather than causation. These findings may inform ongoing efforts to improve equity and efficiency in inpatient resource allocation under China’s evolving health insurance and DRG-based payment reforms.

## Data Availability

The original contributions presented in the study are included in the article/supplementary material, further inquiries can be directed to the corresponding authors.
